# Health Humanities in Medicina: The Auxiliary Stance

**DOI:** 10.3390/medicina58030411

**Published:** 2022-03-10

**Authors:** Olaf Dammann, Signe Mežinska, Eugenijus Gefenas

**Affiliations:** 1 Deptartment of Public Health & Community Medicine, Tufts University School of Medicine, Boston, MA 02111, USA; 2 Department of Gynecology & Obstetrics, Hannover Medical School, D-30635 Hannover, Germany; 3 Department of Neuromedicine and Movement Science, Norwegian University of Science & Technology, NO-7491 Trondheim, Norway; 4 Faculty of Medicine, University of Latvia, LV-1586 Riga, Latvia; signe.mezinska@lu.lv; 5 Center for Health Ethics, Law and History, Institute of Health Sciences, Faculty of Medicine, Vilnius University, LT-03101 Vilnius, Lithuania; eugenijus.gefenas@mf.vu.lt

At the core of medicine is the idea to help fellow human beings by improving or even restoring their health. Let us call this the auxiliary stance of medicine—the motivation of medical intervention by reference to a moral obligation to guide our peers in their attempt to live a healthy and productive life. In parallel, the auxiliary stance is also central to public health, with a focus on prevention and health promotion. Taken together, we can view medicine and public health as the two main human auxiliary endeavors to protect individuals and populations from health risks and help them to heal when sick.

However, medicine and public health are not the only auxiliary human activities. A whole host of other professions are there to help. After all, this is what firefighters, teachers, and bakers do—they help us going through life safely, educated, and well nourished. Along these lines, art, history, literature, philosophy, psychology, religion, and other fields collectively contribute to health and healing in various ways. The intersection of medicine and public health, the health sciences, and the above areas is called health humanities—the concept of infusing medicine and public health in education and practice with ideas from the arts, social sciences, and humanities. The aim of this infusion is not only to constantly reflect on important concepts, such as health, disease and illness, causation, evidence, etc., but also to contribute to defining public health goals, understanding the role of medicine, and building physician-patient relationships.

Specific realms within the health humanities that occupy established spots in academic research communications, for example, medical, and public health ethics, already have their own journals and textbooks. Some journals are devoted to the publication of material from health humanities, e.g., *Philosophy of Medicine, Journal of Medical Humanities, Perspectives in Biology and Medicine, Philosophy, Ethics, and Humanities in Medicine, Medicine, Healthcare and Philosophy* among others. Leading medical publications such as *JAMA* (The Arts and Medicine) and *The Lancet* (Perspectives) provide room for reflection, discussion, and proposals in support of the auxiliary stance.

In keeping with the recognition that such interdisciplinary work can contribute to the mission to help, a new section has been added to Medicina, *Health Humanities*. In this section, we will aim to publish work that reflects on the broader context in which we understand medicine, public health, and the health sciences, written by authors from within or outside the health professions. Our goal is to provide a space for academic papers that are conceived and written with the auxiliary stance in mind, regardless of the academic affiliation of their authors.

For example, interesting and relevant interdisciplinary papers could be those reflecting on ethical and policy making consequences of introducing new regulations and other normative documents. Just to give a couple of examples, in Europe the new Clinical Trials Regulation on Medicinal Products for Human Use has significantly shaped the ethics review of biomedical research in all the EU member states. Similarly, the European General Data Protection Regulation (GDPR) has transformed the understanding and practice of informed consent as it has been defined in traditional research ethics guidelines with important practical consequences for biobanking and big data research [1]. We think these types of papers (combining ethical/social sciences/legal/policy making perspectives) will be relevant for the readership of Medicina. The Health Humanities section will welcome and review contributions in philosophy of medicine and health, history, and ethics of health care, health policy, and communication, medical education, anthropology and other well-established branches of knowledge. Additionally, by no means does the kind of paper we envision to include in this section need to come from the “traditional” areas of interest in the health humanities. Indeed, current work expands into exciting new areas. As discussed by Hedy Wald and colleagues, novel topics for medical humanities include, but are obviously not limited to, global health, informatics, pre-medical education, resilience/wellbeing as well as technological advancements, such as nanotechnology, genome editing, and robotics to mention just a few examples [2].

However, we deliberately want to include research from all health humanities, not just medical. For example, population health has emerged as a topic area to which colleagues from the humanities keep making important contributions. Philosopher Sean Valles has written a comprehensive book entitled *Philosophy of Population Health Science* [3] from which “any population health practitioner or theoretician interested in thinking about meaning, reasoning, and ethics in population health will benefit,” as one of us wrote in a rather enthusiastic review of the book [4]. What strikes us as most interesting is how Valles’ clear commitment to the auxiliary stance is augmented by his suggestion that epistemic, intersectoral, and disciplinary humility would be a good place to start any population health project. Population health has clearly become a focus point of public discussion in the context of the COVID-19 pandemic. For example, philosopher Maya Goldenberg argues in her book *Vaccine Hesitancy* that the rejection of the SARS-CoV-2 immunization by a large segment of the U.S. population is not due to a public misunderstanding of science, but a mistrust of it [5]. In addition, thinking about population health helps us to see the consequences of health inequities and importance of international justice debate. In this respect, recent COVID-19 vaccine distribution controversies that have been described by the WHO as a “catastrophic moral failure” cannot be ignored [6].

We believe that such issues and topics are important for, or should at least be of considerable interest to, the readership of Medicina. We would be delighted to receive your submission to its new section, *Health Humanities*.
